# Dimerization of Tetherin Is Not Essential for Its Antiviral Activity against Lassa and Marburg Viruses

**DOI:** 10.1371/journal.pone.0006934

**Published:** 2009-09-09

**Authors:** Toshie Sakuma, Akira Sakurai, Jiro Yasuda

**Affiliations:** First Department of Forensic Science, National Research Institute of Police Science, Kashiwa, Japan; Institut Pasteur, France

## Abstract

Tetherin (also known as BST2, CD317 or HM1.24) has recently been reported to inhibit a wide range of viruses. However, the antiviral mechanism of action of tetherin has not been determined. Both ends of the tetherin molecule are associated with the plasma membrane and it forms a homodimer. Therefore, a model in which progeny virions are retained on the cell surface by dimer formation between tetherin molecules on the viral envelope and plasma membrane has been proposed as the antiviral mechanism of action of this molecule. To investigate this possibility, we examined the correlation between dimerization and antiviral activity of tetherin in Lassa and Marburg virus-like particle production systems using tetherin mutants deficient in dimer formation. However, the tetherin mutant with complete loss of dimerization activity still showed apparent antiviral activity, indicating that dimerization of tetherin is not essential for its antiviral activity. This suggests that tetherin retains progeny virions on the cell surface by a mechanism other than dimerization.

## Introduction

Tetherin (also known as BST2, CD317, or HM1.24) is a cellular factor identified as HIV-1 release inhibitor [1], [2]. Recently, it has also been reported to show antiviral activity against other retroviruses, filoviruses, and arenaviruses [3], [4]. Tetherin is constitutively expressed in terminally differentiated B cells, bone marrow stromal cells, and plasmacytoid dendritic cells, and is also broadly induced by treatment with IFN-α in various cell types [5], [6]. Therefore, tetherin is thought to be involved in antiviral host defense as an innate immunity mechanism.

Tetherin consists of four domains, *i.e.*, an N-terminal cytoplasmic tail (CT), a single transmembrane domain, an extracellular domain, and a putative C-terminal glycosyl phosphatidylinositol (GPI) anchor, and is present on the cell surface and in perinuclear compartments [5], [7]. Tetherin appears to inhibit virus production by retaining progeny viral particles at the cell surface [1], [2], [4]. However, little is known about the antiviral mechanism of action of tetherin.

Tetherin is anchored in the cell membrane at both ends and forms a homodimer [7], [8]. Therefore, it has been suggested that tetherin retains progeny virions at the cell surface by dimerization between tetherin monomers in apposing cellular and viral membranes [1], [9]. Although this model seems very likely, no supporting evidence has yet been reported.

The tetherin dimer appears to be formed by disulfide linkage, as the dimer observed under nonreducing conditions is disassembled to monomers under reducing conditions [8]. Human tetherin contains five cysteine residues. Three conserved cysteine residues are present in the extracellular domain at positions 53, 63, and 91, and they are considered to be involved in disulfide-linked dimer formation [7], [8].

Lassa virus and Marburg virus cause hemorrhagic fever in humans and nonhuman primates, and it is necessary to develop therapeutic strategies and vaccines to prevent viral infections. Previously, we showed that tetherin inhibits the egress of Marburg and Lassa viral particles using the system for producing virus-like particles (VLP) induced by expression of viral matrix proteins [4].

To determine its antiviral mechanism of action, we examined the correlation between dimerization and antiviral activity of tetherin. In addition, we also discuss possible mechanisms for the antiviral function of tetherin.

## Results and Discussion

### Is dimerization of tetherin required for its antiviral activity?

**Figure 1:**
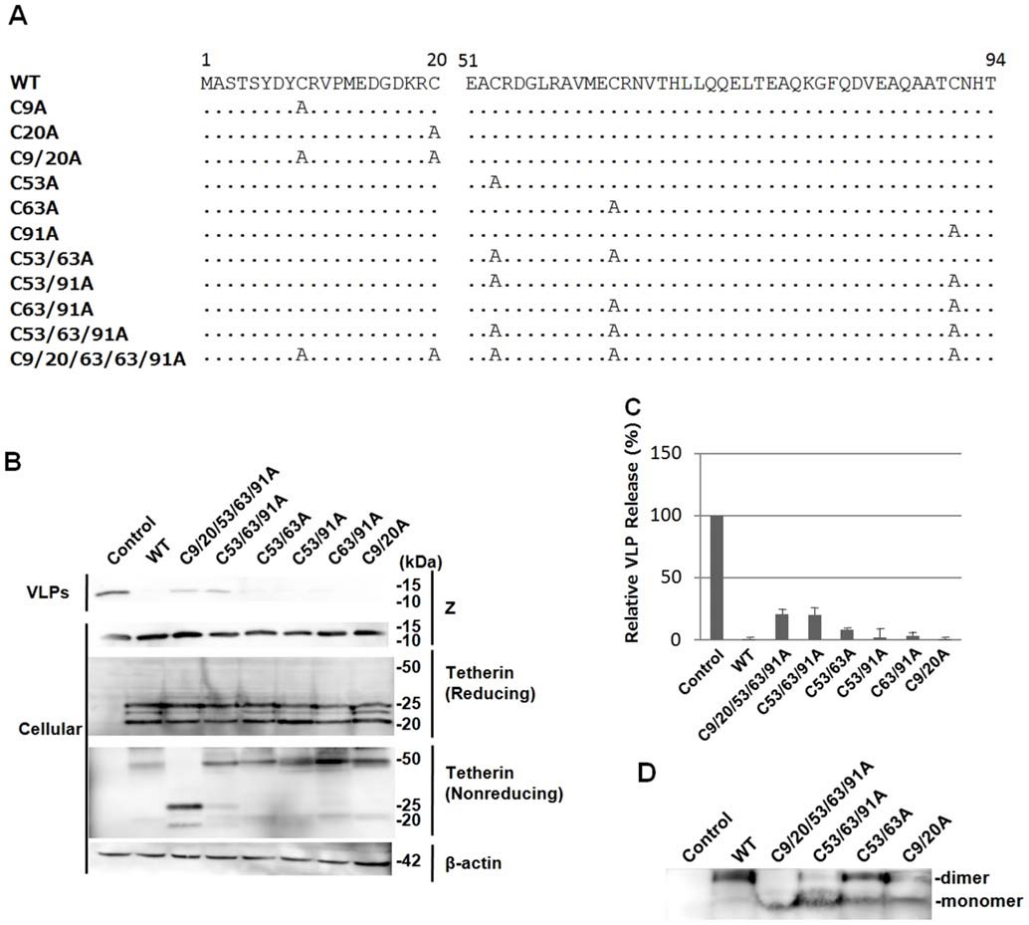
Correlation between dimerization and antiviral activity of tetherin. (A) Human tetherin has two cysteine residues in the N-terminal intracellular domain, C9 and C20, and three cysteine residues in the extracellular domain, C53, C63, and C91. Various cysteine to alanine mutants were constructed as described in Materials and Methods. (B) Lassa virus Z protein was coexpressed with wild-type (WT) or mutant tetherin in COS-7 cells. VLP-associated and cell-associated Z were analyzed by Western blotting. WT and mutant tetherin expressed in COS-7 cells were analyzed under both reducing and nonreducing conditions by Western blotting. Western blotting for β-actin was performed as an internal control. (C) The intensities of the bands for VLP-associated Z in panel B were quantified using a LAS3000 imaging system (Fuji Film, Tokyo, Japan). The level of Z in VLP released from cells cotransfected with control vector was set to 100%. The data are shown as averages and standard deviations (SD) of 6 independent experiments. (D) Dimerization of WT or mutant tetherin expressed in COS-7 cells was also analyzed by Native-PAGE.

**Figure 2:**
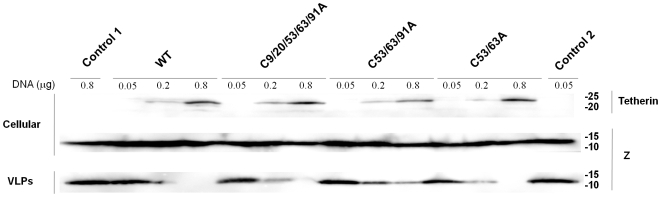
Dose-dependent reduction of VLP release at increasing expression levels of tetherin. COS-7 cells were transfected with 0.5 µg of the expression plasmid for Lassa Z, pCLV-Z, together with the empty vector (Control 1: 0.8 µg, Control 2: 0.05 µg) or 0.05, 0.2 and 0.8 µg of plasmid DNA for WT or mutant tetherin. VLP-associated and cell-associated Z were analyzed by Western blotting. WT and mutant tetherin expressed in COS-7 cells were analyzed under reducing conditions by Western blotting.

Human tetherin possesses five cysteine residues, three in the extracellular domain and two in the intracellular domain. Three cysteine residues in the extracellular domain, C53, C63, and C91, are conserved among human, rhesus monkey, rat, and mouse, while two cysteine residues in the intracellular domain, C9 and C20, are conserved only between human and rhesus monkey [7], [8]. It is very likely that disulfide bond formation by these cysteine residues is involved in dimerization of tetherin. To examine whether the dimerization of tetherin is important for its antiviral activity, we first generated tetherin mutants with cysteine to alanine substitutions (Figure 1A) and analyzed the effects of exogenous expression of these mutants on release of Lassa Z-induced VLP. The Lassa Z expression plasmid was cotransfected into COS-7 cells with the expression plasmid for wild-type (WT) or mutant tetherin. As expected, WT tetherin was detected as a dimer of around 50 kDa under nonreducing conditions and as triplet bands of monomer of around 20–25 kDa under reducing conditions (Figure 1B). Single and double substitutions at C9, C20, C53, C63, and C91 had little effect on dimer formation of tetherin (data not shown for single mutants). In the C53/63/91A mutant in which all three cysteine residues in the extracellular domain were replaced with alanine, dimer formation was partially disrupted. The dimerization of tetherin was almost completely abolished in the C9/20/53/63/91A mutant in which all five cysteine residues were substituted with alanine, indicating that these cysteine residues are involved in dimer formation. As shown in Figures 1B and C, the tetherin mutants with double substitutions inhibited the production of Lassa Z-induced VLP to a similar level as WT tetherin. Moreover, the C53/63/91A, which showed reduced dimerization, and the C9/20/53/63/91A, which was almost completely disrupted dimerization, still inhibited Lassa VLP production despite the possibility that the introduction of alanine mutations into multiple cysteine residues may also disrupt the tertiary structure of tetherin. We also analyzed dose-dependent reductions of VLP release at increasing expression levels of WT and mutants of tetherin. As shown in Figure 2, dose-dependent reductions of VLP release were observed in cells expressing WT and mutants of tetherin. The C53/63A mutant showed similar activity to WT in the inhibition of VLP release, while the C53/63/91A and C9/20/53/63/91A mutants showed a little reduced activity. This is consistent with the results from Figure 1B and C. Although the level of dimerization of the C9/20/53/63/91A mutant is significantly different from that of the C53/63/91A mutant, inhibitory activities of these mutants on VLP release were very similar. Therefore, the reduced inhibitory activities of C53/63/91A and C9/20/53/63/91A mutants may be because the tertiary structure of tetherin was affected by the cysteine to alanine mutations at multiple sites, but not due to loss of dimerization activity of tetherin. Thus, these results indicated that dimerization by intermolecular disulfide bonds is not essential for the antiviral activity of tetherin.

The dimer of tetherin could be also formed by intermolecular interaction other than disulfide bonds. To examine this possibility, we further analyzed the dimerization of tetherin mutants by Native-PAGE (Figure 1D). The analysis by Native-PAGE also showed that most forms of the C9/20/53/63/91A mutant in cells are monomer, but not dimer.

Taken together, these results indicated that the dimerization of tetherin is not essential for its inhibitory activity against Lassa VLP release.

**Figure 3:**
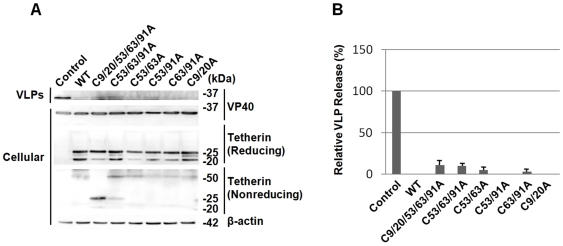
Effects of expression of the cysteine to alanine substitution mutants of tetherin on Marburg VLP production. (A) Marburg virus VP40 protein was coexpressed with WT or mutant tetherin in COS-7 cells. VLP-associated and cell-associated VP40 were analyzed by Western blotting. WT and mutant tetherin expressed in COS-7 cells were analyzed under both reducing and nonreducing conditions by Western blotting. Western blotting for β-actin was performed as an internal control. (C) The intensities of the bands for VLP-associated VP40 in panel A were quantified using a LAS3000 imaging system (Fuji Film). The level of VP40 in VLP released from cells cotransfected with control vector was set to 100%. The data are shown as averages and standard deviations (SD) of 6 independent experiments.

Similar results were also obtained when Marburg matrix protein VP40 was coexpressed with WT or mutant tetherin (Figure 3A and B). The production of Marburg VP40-induced VLP from cells expressing VP40 was strongly inhibited by WT or any mutant of tetherin. These results indicated that dimerization of tetherin is not essential for its antiviral activity.

### Intracellular localization of tetherin and viral proteins

**Figure 4:**
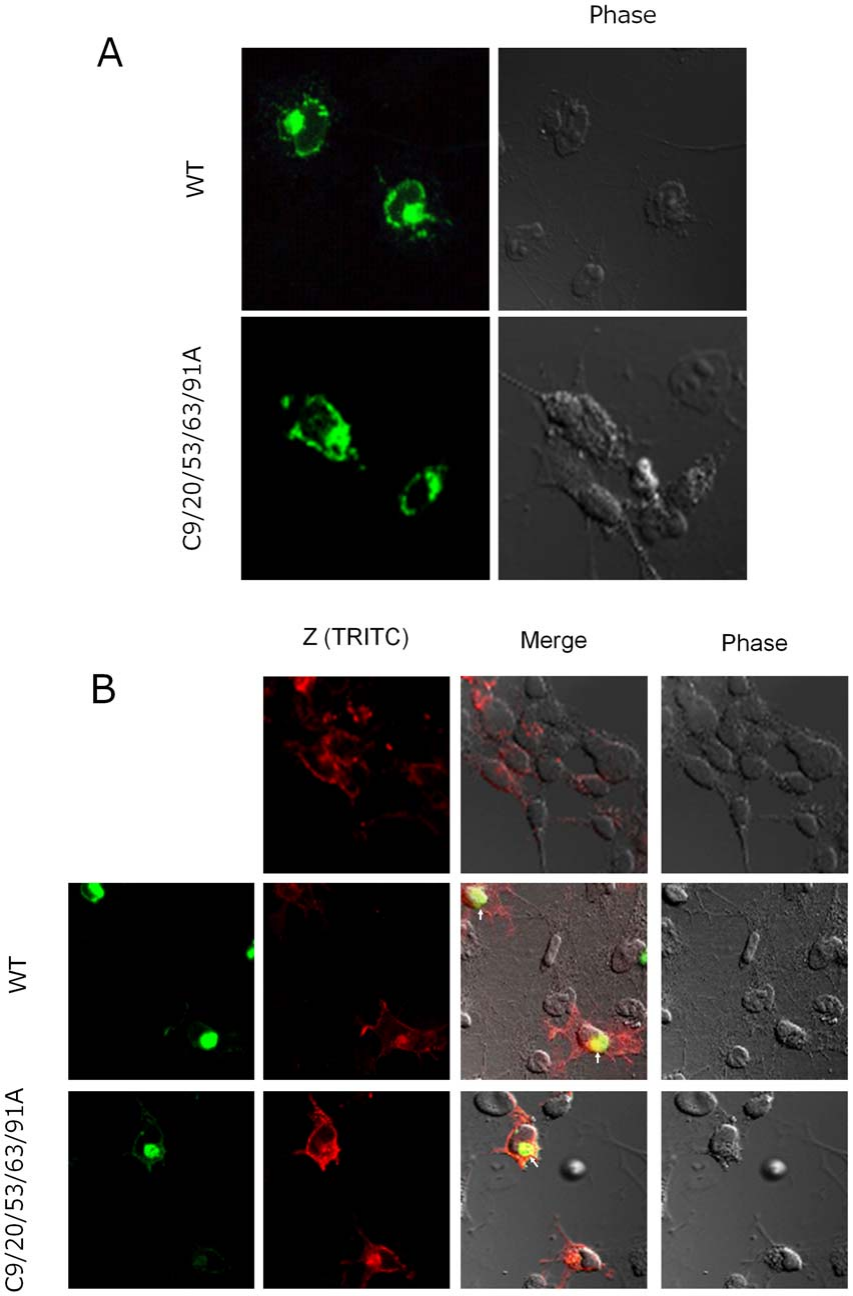
Intracellular localization of Lassa virus Z protein and WT or mutant tetherin. (A) COS-7 cells expressing WT or C9/20/53/63/91A mutant tetherin were fixed and processed for immunofluorescence analysis as described in Materials and Methods. (B) The expression plasmid for Lassa Z, pCLV-Z, was cotransfected with the expression plasmid for WT or C9/20/53/63/91A mutant tetherin in COS-7cells. At 48 h posttransfection, staining was performed for WT and mutant tetherin and Z as described in Materials and Methods. WT and mutant tetherin, green; Z, red.

We next examined the intracellular localization of WT and mutant tetherin by immunofluorescence confocal microscopy (Figure 4). As shown in Figure 4A, WT tetherin was mainly localized around the perinuclear region and the plasma membrane as dispersed dots. These observations were consistent with those reported previously [1], [2], [5], [7]. The C9/20/53/63/91A mutant also showed similar intracellular localization to WT. Lassa Z protein was found in the plasma membrane and cytoplasm when expressed alone (Figure 4B upper). When Lassa Z protein and WT or mutant tetherin were coexpressed in cells, WT and mutant tetherin were mainly localized to a patch in the cytoplasm (Figure 4B middle and lower). In addition, WT and mutant tetherin showed partial colocalization with Lassa Z protein in a small region within the cytoplasmic patch of tetherin (Figure 4B middle and lower: arrowheads).

Although Neil *et al*. [1] and Van Damme *et al*. [2] previously showed that HIV-1 Gag colocalizes with tetherin in the plasma membrane and endosome, colocalization of tetherin and Lassa Z protein in the plasma membrane was not seen only partial in our experiment using the Lassa Z-induced VLP production system. Retention of viral protein by tetherin in cytoplasmic organelles, such as the endosome, may be also involved in the antiviral mechanism of action of this molecule.

### Proposed models for the antiviral function of tetherin

**Figure 5:**
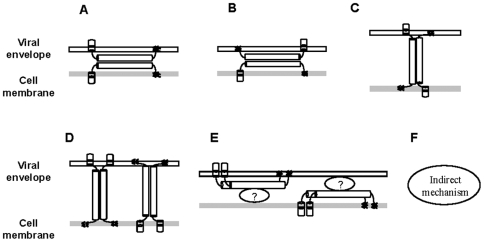
Proposed models for antiviral mechanism of action of tetherin.

Here, we propose an antiviral mechanism of action of tetherin based on the findings of the present study. As shown in Figure 5A–F, there are several possible models. Models A and B, which require dimerization of tetherin for its antiviral function, were proposed previously [9]. However, these models appear not to be major mechanisms involved in the antiviral function of tetherin, as our findings showed that dimerization of tetherin is not essential for its antiviral activity. Neil *et al*. reported that truncation of the N-terminal cytoplasmic region or removal of the C-terminal GPI anchor completely abolished the antiviral activity of tetherin [1]. We have also confirmed that the tetherin mutant without the GPI anchor showed complete loss of the antiviral activity (data not shown). In Model C, tetherin retains virions on the cell surface by dimerization between tetherin monomers on apposing cellular and viral membranes, even though tetherin does not have a GPI anchor. This model is incorrect as C-terminal GPI modification of tetherin is essential for its antiviral activity. In contrast, in Model D, the GPI anchor is essential for retention of virions independent of dimer formation of tetherin. Thus, Model D, but not Models A, B, or C, is the most likely, if tetherin solely retains virions on the cell surface.

As shown in Model E, tetherin may retain virions by interaction with other cellular or viral components on the cell membrane or viral envelope. Kaletsky *et al*. recently reported that Ebola GP can interact directly with tetherin and antagonize its antiviral activity [10]. However, cell surface retention of virions by this interaction with viral GP appears not to be the antiviral mechanism of action of tetherin, as tetherin can also retain VLP induced by only VP40 [4], [10]. VP40 is viral matrix protein present inside the virion and is not presented outside the viral envelope. Therefore, it is unlikely that tetherin interacts directly with VP40 on the cell surface. This is also supported by the observation that colocalization of tetherin and Ebola VP40 or Lassa Z is only partial or not significant (Figure 4) [10]. Tetherin on the viral envelope or cell membrane would interact with cellular factors on the cell membrane or viral envelope, respectively. To demonstrate whether this model is correct, it will be necessary to identify the cellular factor(s) with which tetherin interacts.

Although we proposed a model in which tetherin directly retains virions on the cell surface, it may indirectly inhibit virion release from cells (Model F in Figure 5). The observation that colocalization of tetherin and viral protein on the cell surface was not significant despite accumulation of large numbers of virions on the cell surface may support this model (Figure 4) [4].

Taken together, Models D, E and F remain possible as mechanisms of the antiviral effect of tetherin. Further analyses are required to clarify the antiviral mechanism of action of tetherin.

Furthermore, electron microscopic analyses showed that tetherin appears to tether virions to each other as well as to the cells [1], [4]. This virion to virion attachment by tetherin may also be why tetherin effectively inhibits virus release.

## Materials and Methods

### Plasmid construction

Plasmids for expression of Lassa Z or Marburg VP40, pCLV-Z and pMV-VP40, respectively, were described previously [11], [12]. An expression plasmid for WT human tetherin with a FLAG-tag at the N-terminus, pTeth-FL, was constructed previously [4]. Tetherin mutants with cysteine to alanine substitution(s), C53A, C63A, C91A, C53/63A, C53/91A, C63/91A, C53/63/91A, and C9/20/53/63/91A, were generated from pTeth-FL using a QuikChange II site-directed mutagenesis kit (Stratagene, La Jolla, CA, USA) (Figure 1A).

### VLP release assay

COS-7 cells were maintained in Dulbecco's modified Eagle's medium supplemented with 10% fetal calf serum at 37°C in 5% CO_2_. VLP release assay was carried out as described previously [4], [11]–[14]. Briefly, pCLV-Z (0.5 µg) or pMV-VP40 (0.1 µg) was cotransfected with 0.8 µg of the expression plasmid for WT or mutant tetherin into COS-7cells. At 48 hours after transfection, VLP in the cell supernatants were collected by ultracentrifugation and then analyzed by Western blotting. Cells were lysed with TNE buffer containing 10 mM Tris-HCl (pH 7.8), 0.5% NP40, 0.15 M NaCl, and 1 mM EDTA, and then treated with SDS-PAGE sample loading buffer either with or without 2-mercaptoethanol for the reduced or nonreduced form, respectively. Both reduced and nonreduced cell lysates were separated by SDS-PAGE and then analyzed by Western blotting as described previously [4].

### Native PAGE

COS-7 cells were transfected with 0.8 µg of the expression plasmid for WT or mutant tetherin. At 48 hours after transfection, cells were lysed with the NativePAGE sample buffer containing 1% Digitonin (Invitrogen, Carlsbad, CA, USA). Cell lysates were separated by Blue Native PAGE [15] and then analyzed by Western blotting using anti-FLAG antibody as described previously [4].

### Immunofluorescence microscopy

At 48 hours posttransfection, COS-7 cells were fixed in 4% formaldehyde. The fixed cells were treated with 0.1% Triton X-100 for 15 min. Mouse anti-FLAG monoclonal antibody or rabbit anti-Z antibody was used to stain FLAG-tagged tetherin or Lassa Z, respectively, followed by goat anti-mouse IgG conjugated with FITC or goat anti-rabbit IgG conjugated with TRITC, respectively. Cells were observed by confocal microscopy LSM5Pascal (Carl Zeiss, Oberkochen, Germany).

## References

[1] Neil SJD, Zang T, Bieniasz PD (2008). Tetherin inhibits retrovirus release and is antagonized by HIV-1 Vpu.. Nature.

[2] Van Damme N, Goff D, Katsura C, Jorgenson RL, Mitchell R (2008). The interferon-induced protein BST-2 restricts HIV-1 release and is downregulated from the cell surface by the viral Vpu protein.. Cell Host Microbe.

[3] Jouvenet N, Neil SJD, Zhadina M, Zang T, Kratovac Z (2009). Broad-spectrum inhibition of retroviral and filoviral particle release by tetherin.. J Virol.

[4] Sakuma T, Noda T, Urata S, Kawaoka Y, Yasuda J (2009). Inhibition of Lassa and Marburg virus production by tetherin.. J Virol.

[5] Blasius A L, Giurisato E, Cella M, Schreiber RD, Shaw AS (2006). Bone marrow stromal cell antigen 2 is a specific marker of type I IFN-producing cells in the naive mouse, but a promiscuous cell surface antigen following IFN stimulation.. J Immunol.

[6] Ishikawa J, Kaisho T, Tomizawa H, Lee BO, Kobune Y (1995). Molecular cloning and chromosomal mapping of a bone marrow stromal cell surface gene, BST2, that may be involved in pre-B-cell growth.. Genomics.

[7] Kupzig S, Korolchuk V, Rollason R, Sugden A, Wilde A (2003). Bst-2/HM1.24 is a raft-associated apical membrane protein with an unusual topology.. Traffic.

[8] Ohtomo T, Sugamata Y, Ozaki Y, Ono K, Yoshimura Y (1999). Molecular cloning and characterization of a surface antigen preferentially overexpressed on multiple myeloma cells.. Biochem Biophys Res Commun.

[9] Göttlinger HG (2008). Virus kept on a leash.. Nature.

[10] Kaletsky RL, Francica JR, Agrawal-Gamse C, Bates P (2009). Tetherin-mediated restriction of filovirus budding is antagonized by the Ebola glycoprotein.. Proc Natl Acad Sci USA.

[11] Urata S, Noda T, Kawaoka Y, Yokosawa H, Yasuda J (2006). Cellular factors required for Lassa virus budding.. J Virol.

[12] Urata S, Noda T, Kawaoka Y, Morikawa S, Yokosawa H (2007). Interaction of Tsg101 with Marburg virus VP40 depends on the PPPY motif, but not the PT/SAP motif as in the case of Ebola virus, and Tsg101 plays a critical role in the budding of Marburg virus-like particles induced by VP40, NP, and GP.. J Virol.

[13] Yasuda J, Hunter E, Nakao M, Shida H (2002). Functional involvement of a novel Nedd4-like ubiquitin ligase on retrovirus budding.. EMBO Rep.

[14] Yasuda J, Nakao M, Kawaoka Y, Shida H (2003). Nedd4 regulates egress of Ebola virus-like particles from host cells.. J Virol.

[15] Schagger H, von Jagow G (1991). Blue Native electrophoresis for isolation of membrane protein complexes in enzymatically active form.. Anal Biochem.

